# SYMPTOM EXPERIENCES OF HOSPITALIZED OLDER ADULTS ON ADMISSION AND FUNCTIONAL OUTCOMES 1 MONTH POST-DISCHARGE

**DOI:** 10.1093/geroni/igz038.1676

**Published:** 2019-11-08

**Authors:** Anna Zisberg, Ksenya Shulyaev, Anat Liberty, Nurit Gur-Yash, Maayan Agmon, Dorit Pud

**Affiliations:** 1 The Cheryl Spencer Department of Nursing Faculty of Social Welfare and Health Science, University of Haifa, Israel, Haifa, Isreal, Israel; 2 The Cheryl Spencer Department of Nursing Faculty of Social Welfare and Health Science, University of Haifa, Mount Carmel, Israel, Haifa, Israel, Israel; 3 HaEmek Medical Center, Clalit Health Services, Afula, Israel, Afula, Israel, Israel; 4 Oranim, College of Educational, Israel., Oranim, Israel

## Abstract

The aims of this study were to identify subgroups of older adults admitted to hospital based on their experience with multiple symptoms and to explore if these subgroups differed on physical and cognitive characteristics at time of admission, and on functional outcomes one-month post-discharge. The study included 331 older adults (mean age 75.5±7.1), admitted to hospital and were hospitalized in internal-medicine units. Demographic, functional, cognitive, psychological and mobility characteristics and symptoms’ disturbance were assessed within the first 24 hours of admission and one moth following discharge. Cluster analysis identified three distinct subgroups based on patients’ experiences with five highly prevalent symptoms (tiredness, dyspnea, dizziness, sleep disturbance and pain): Low or high levels of all five symptoms (70%, 14%, respectively), and moderate levels of four symptoms with high dyspnea (14%). “All high” cluster was characterized by the worst cognitive and instrumental function, and highest anxiety and depression levels. The “moderate with high dyspnea” subgroup expressed the highest comorbidity score. Multivariate Logistic regression showed that the odds of decline in instrumental ADL one month post-discharge was 3.28 (95% CI 3.21-3.25, p=.021) for “all high” and 2.35 (95% CI 2.33-2.36, p=.043) for “all low” symptom-subgroups compared to “medium with high dyspnea” subgroup adjusted for pre-morbid function, health conditions and demographic characteristics. Belonging to certain symptom-subgroups is an important risk factor in predicting negative consequences of hospitalization. These findings emphasize the importance of evaluating and subgrouping broad range of symptoms among hospitalized older adults.

